# Folic Acid Levels During Pregnancy Regulate Trophoblast Invasive Behavior and the Possible Development of Preeclampsia

**DOI:** 10.3389/fnut.2022.847136

**Published:** 2022-04-28

**Authors:** Beenish Rahat, Abid Hamid, Rashmi Bagga, Jyotdeep Kaur

**Affiliations:** ^1^Department of Biochemistry, Postgraduate Institute of Medical Education and Research, Chandigarh, India; ^2^Cancer Pharmacology Division, CSIR-Indian Institute of Integrative Medicine, Jammu, India; ^3^Department of Obstetrics and Gynecology, Postgraduate Institute of Medical Education and Research, Chandigarh, India

**Keywords:** folate (folic acid), trophoblast invasion, preeclampsia, pregnancy, DNA methylation, placenta, DNMTs, matrix metalloproteinases

## Abstract

**Background:**

One of the unique features of placentation is its similarity to tumorigenesis yet being very well regulated. It allows rapid proliferation, migration, and invasion of mononuclear trophoblast cells into the maternal uterus and remodeling the maternal vasculature. This pseudomalignant nature of trophoblastic cells is strictly regulated and its importance becomes evident in abnormal pregnancies that are characterized by aberrant trophoblast proliferation/invasion like preeclampsia. In addition to this, the importance of folic acid supplementation during pregnancy is well documented. We aimed to analyze the molecular and epigenetic regulation of the pseudomalignant nature of placentation *via* folic acid levels.

**Methods:**

Placental tissue samples were collected from different pregnancies in three different gestational stages. We estimated the impact of folic acid levels on global methylation, *LINE1* methylation, and expression of DNMTs in all three gestational stages in pregnant women and preeclampsia pregnancies. We also analyzed the effect of folic acid supplementation on trophoblastic invasion using placental derived cells viz, JEG-3 and HTR-8/SVneo cell line and verified the molecular and epigenetic mechanisms involved in this regulation.

**Results:**

Development of preeclampsia was observed to be associated with lower folate levels in placental tissue, higher global methylation level, and higher expression of *DNMT1*and *DNMT3A*. Folic acid supplementation was found to increase the invasive potential of placental trophoblasts by almost two folds which were associated with the decreased expression of tumor suppressor genes and tissue inhibitors of matrix metalloproteinases; and increased expression of oncogenes, telomerase gene, and matrix metalloproteinases. These folic acid-mediated changes were observed to be regulated by CpG methylation in the case of many genes. Folic acid supplementation was also observed to significantly decrease global methylation in placental trophoblasts related to decreasing expression of *DNMT1* and *DNMT3A*.

**Conclusion:**

Lower folic acid levels are associated with preeclampsia development and folic acid supplementation regulates the invasive potential of placental trophoblasts as mediated by various epigenetic changes in the placenta suggesting the protective effect of folic acid against preeclampsia.

## Introduction

The development of the placenta involves a complex structural and physiological reorganization involving rapid proliferation, migration, invasion of mononuclear trophoblast cells into the maternal uterus, and remodeling of the spiral arteries like most aggressive tumors (1). Regulation of placental development involves a highly complex differential global gene expression profile varying with gestational age. This pseudomalignant nature of trophoblastic cells is strictly regulated. The placenta is, therefore, considered a “well-behaved” tumor (2). The importance of these intrinsic molecular controls becomes increasingly evident in abnormal pregnancies that are characterized by aberrant trophoblast proliferation/invasion like preeclampsia.

Our previous studies have shown the role of epigenetic mechanisms in regulating the expression of various sets of genes like tumor suppressors, oncogenes, telomerase, extracellular matrix digesting and their inhibitors, and WNT signaling inhibitors as contributing factors in maintaining normal pregnancy and dysregulation of these mechanisms behind the development of preeclampsia (3–6). WNT signaling modulates the invasiveness of human trophoblasts and this pathway is regulated by some specific antagonists like Adenomatosis Polyposis Coli (APC), Dickkopf1 (Dkk1), and WNT inhibitory factor 1 (WIF-1). These three constitute inhibitors of WNT signaling and among these APC plays the most important role in placental development (7). Folic acid is involved in DNA synthesis, acts as a methyl donor in DNA and histone methylation processes and hence plays a role in epigenetic mechanisms, and has the potential to alter gene expression. Folic acid has special importance in pregnancy aiding in rapid cell division and growth. The US National Institutes of Health (NIH) and the U.S. Public Health Service have recommended a daily supplementation of 400–600 μg of folic acid by pregnant women (8, 9). Folic acid supplementation has been widely prescribed to pregnant women to prevent neural tube closure defects in newborns and the additional beneficial effects on the neurodevelopment of children (8, 10). Although neural tube closure occurs within the first trimester, high doses of folic acid are given throughout pregnancy, the physiological consequences of which are unknown.

Further, folic acid supplementation is known to affect the transcription of various genes, with both enhancing and down regulating gene expression (11, 12). is an association with its promoting or repressing effects on DNA and histone methylation (13, 14). A recent review has summarized the link between DNA methylation, maternal folic acid supplementation, and offspring health (15). Li et al. (16) has related folic acid supplementation with improved insulin resistance by inducing DNA methylation changes (16).

The use of supplemental folic acid is widespread, however, there is no information on the effect of folate supplementation on the epigenetic and molecular regulation of various genes in the placenta. A study has shown the potential effects of folic acid to affect the trophoblasts in placental explants (17). A recent study has also shown the negative effect of excess folic acid treatment in the villous trophoblast (18). Excess folate causing saturation of dihydrofolate reductase (DHFR) in maternal and fetal circulation, has been linked to the long-term health outcomes of the offspring (19–21). Thus, a detailed study on the effects of folic acid supplementation on trophoblastic invasion under different conditions would be informative.

Our previous study has reported the role of lower folate levels in the development of preeclampsia (22). A 2018 meta-analysis has also related folate supplementation with reduced risk of preeclampsia (23). Hence, we designed this study to analyze the effect of folic acid levels on the different epigenetic phenomena during different trimesters in normal pregnancy and their role in the development of preeclampsia. Further, we estimated the effect of folic acid supplementation on trophoblastic invasion and the molecular and epigenetic regulation behind it. Pregnant women were divided into four groups, based on the three trimesters in normal pregnancy and preeclampsia. The placental villi sample was obtained from each pregnant woman. Folic acid supplementation study was done in *in vitro* in placental derived cells *viz*, JEG-3 and HTR-8/SVneo cell lines.

## Materials and Methods

### Ethical Approval

This study was carried out in the department of Biochemistry in collaboration with the Department of Obstetrics and Gynecology at the Postgraduate Institute of Medical Education and Research (PGIMER), Chandigarh, India. The protocol of the study was approved by the Institute Ethics Committee (IEC) and written informed consent was obtained from all participants.

### Subjects and Sample Collection

Pregnant women with the clinical diagnosis of normal pregnancy or pregnancy complicated with preeclampsia attending the Department of Obstetrics and Gynecology were included in this study. Pregnant women with normal pregnancy were divided into three groups based on the gestational age (first, second and third trimester, *n* = 30 in each group), while the preeclampsia group (*n* = 30) was characterized by clinical symptoms of systolic pressure 140 mmHg and diastolic pressure 90 mmHg, proteinuria 300 mg in 24 hr. Placental villi samples were collected after elective pregnancy terminations from pregnant women with normal first and second-trimester pregnancy, or after cesarean section or vaginal deliveries in case of normal third-trimester and pre-eclamptic pregnancies. Placental tissue obtained from each subject was processed in chilled PBS for the separation of clear placental villi free from fibroid tissue, blood clots, amnion, and basement membrane.

### Cell Culture

Two adherent cell lines, JEG-3, and HTR-8/SVneo [Source: American Type Culture Collection (ATCC), kindly provided by Dr S.K. Gupta, National Institute of Immunology, New Delhi, India] was used in this study. JEG-3 is placental choriocarcinoma-derived cell line, while HTR-8/SVneo is a cell line derived from first trimester transformed cells and acts as a close mimic of extra-villous trophoblasts. JEG-3 and HTR-8/SVneo cells were cultured in DMEMHG, with 4,500 mg/L glucose and RPMI-1640 medium, respectively, supplemented with L-glutamine and 25 mM HEPES, 10% FBS, sodium bicarbonate (3.7 and 2 g/L, respectively), penicillin (100 U/ml), streptomycin (100 mg/ml) and amphotericin B (0.25 mg/ml) under standard conditions (37°C, 5% CO_2_, humidified atmosphere). Cell culture media and other reagents were purchased from Sigma Chemical Co.

### Estimation of Folate Levels by Microbiological Assay

The folate levels within placental villi samples and cell lines were estimated using the *Lactobacillus casei* assay, as previously explained (24). Briefly, 10% homogenate of villous tissue was prepared in folate extraction buffer containing 50 mM ascorbic acid. The homogenate was incubated for 10 min at 110 °C, followed by centrifugation and the treatment of separated supernatant with rat plasma conjugase, which hydrolyzes the polyglutamated folate to monoglutamated folate form. The free folate is utilized by *L. casei* for its growth; hence the levels of folic acid levels are determined by measuring bacterial growth. In each assay, the standards of known folic acid concentration were run along with the samples.

### Global Methylation

The overall degree of methylation of a genome is a useful measure of global methylation changes. Global DNA methylation was measured using an ELISA based kit Imprint^R^ Methylated DNA Quantification Kit (Sigma-Aldrich, Inc) according to the manufacturer's instructions. Briefly, the methylated DNA is detected using the capture and detection antibodies and then quantified colorimetrically. The amount of methylated DNA present in the sample is proportional to the absorbance measured. To calculate methylation levels relative to the methylated control DNA, we used the single point method, which gives the per cent methylation relative to the methylated control DNA.

### Invasion Assay

An invasion assay was performed to study the invasive property of JEG-3 and HTR-8/SVneo *in vitro* and the effect of folic acid treatment on the invasion of these cell lines. It was done by using the BD Matrigel™ Matrix-GFR (Growth Factor Reduced), which is a reconstituted basement membrane. The Matrigel matrix layer occludes the pores of the PET membrane, blocking non-invasive cells from migrating through the membrane. Exogenous folic acid (10^−7^ M and 10^−4^ M) was added to the culture media, which originally contained no folic acid. 10^−7^ M concentration was chosen because it represents folic acid levels in the physiological ranges and 10^−4^ M represents a much higher concentration than the physiological range (17). 10^−4^ M was chosen to compare the effect of higher folate levels with physiological levels. Cells were incubated for 48 h at 37°C, 5% CO_2_, humidified atmosphere. The invasive cells were able to detach themselves from and invade through the Matrigel matrix and the 8-micron membrane pores. The number of cells invading the Matrigel were counted, after being stained with Hoechst 33342 nuclear-fluorescent stain under Olympus BX61 epi automated fluorescence microscope under 460–90 nm and 40× magnification. The fold change was calculated with respect to the untreated control and the statistical analysis was done by comparing the means of the control and experimental sets by using paired Student's *t*-test.

### Quantitative Real-Time PCR

Total RNA was isolated from placental villi, the JEG-3 and HTR-8/SVneo cell lines using TRIzol (Ambion, Life Technologies Corporation, CA, USA), and 1 mg was reverse-transcribed using RevertAidTM M-MuLV-RT kit (MBI Fermentas, Life Sciences, USA). The resulting cDNA was quantified using Applied Biosystems StepOnePlusTM Real-Time PCR System. The mRNA expression levels of glyceraldehyde-3- phosphate dehydrogenase (GAPDH) were used as the endogenous control. The comparative Ct method (DDCT) (25) was used for the quantification of the transcripts. Measurement of DCt was performed in triplicate.

### Methylation-Sensitive High-Resolution-Melting

DNA was extracted for methylation analysis from the placental villi and cultured JEG-3 and HTR-8/SVneo cell lines by using the genomic DNA isolation kit (Real Genomics, Real Biotech Corporation, Taipei, Taiwan). Promoter region CpG methylation analysis of the target genes was carried out by methylation-sensitive high-resolution melting (MSHRM) as previously explained using gene-specific primers targeting promoter region CpG islands (3–6). Briefly, MS-HRM can differentiate sequences on the basis of the GC content, which determines the temperature at which the dsDNA sequence denatured (26), using a standard curve comprising 0, 0.5, 5, 10, 20, 40, 60, 80, and 100% methylation standards. The methylation percentage values for each target sample were calculated according to the method of Migheli et al. (27) by imputing the average aligned fluorescence value of each sample in the Polyfit interpolation curve (MatLab program-The MathWorks, Inc., USA) (27).

### Statistical Analysis

All statistical analyses were performed using the SPSS software for Windows version 16.0 and GraphPad Prism software version 5.00.288. Between-group comparisons were made using one-way ANOVA; if found to be significant, the Fisher *post-hoc* test was applied. Student's *t*-test and the Mann–Whitney U-test were used to analyzing the data between two variables. Pearson's correlation analysis was used to estimate the correlations between different parameters. The effect of different epigenetic mechanisms on mRNA expression levels was assessed by multiple regression analysis. All statistical tests were two-sided, and differences were considered statistically significant at *p* < 0.05. Unless otherwise stated, all data are expressed as a mean (SEM).

## Results

**Figure 1:**
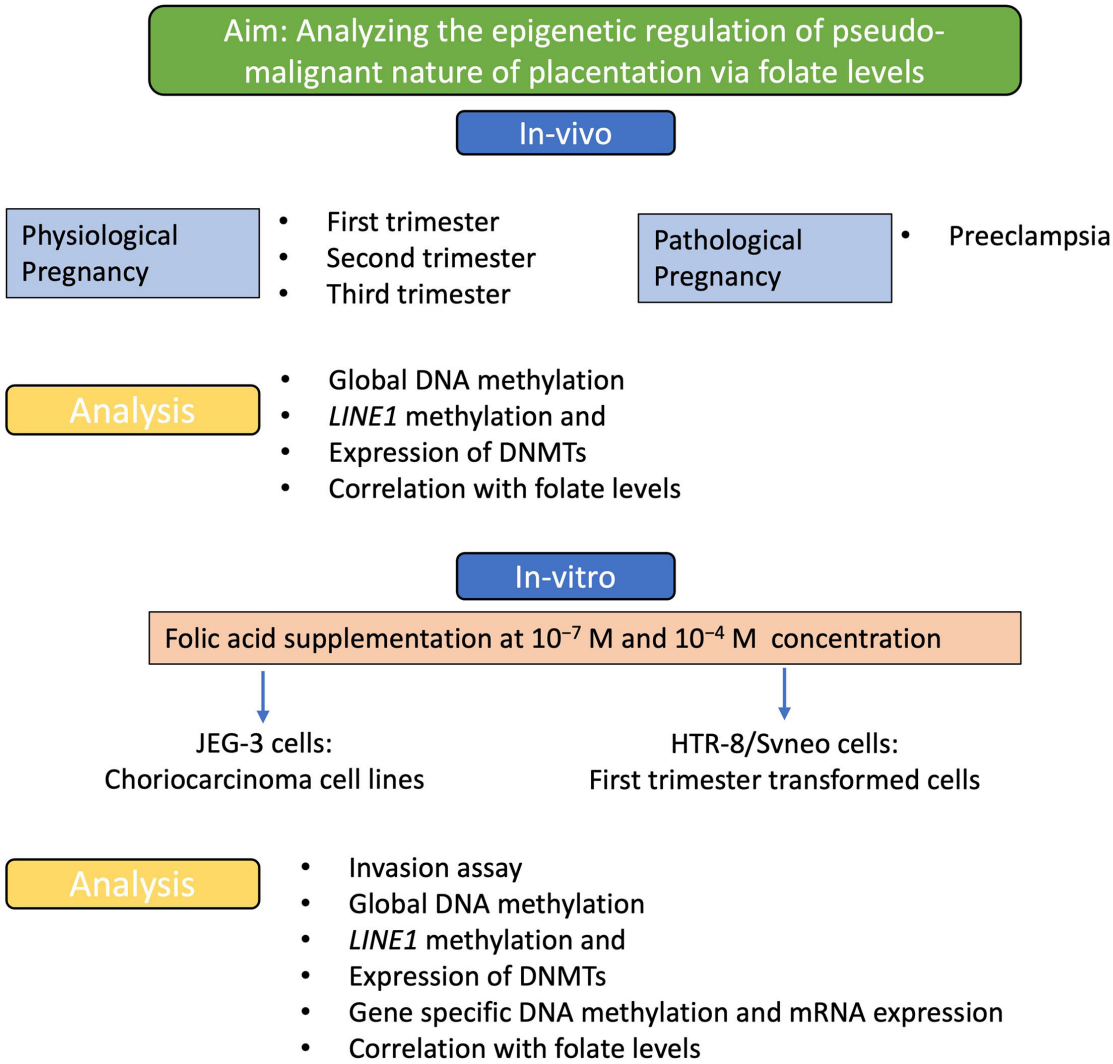
Experimental strategy. A flow diagram to show the aim of the study and the experimental strategies used to achieve this aim. Analyzing the effect of folic acid levels *in-vivo* and the effect of folic acid supplementation *in-vitro*, to understand the effect on the pseudo-malignant phenotype at the molecular and epigenetic levels.

The aim of the study and the different experimental strategies used is demonstrated in the flow diagram shown in Figure 1. The present study was carried out to find the role of folic acid in modulating various epigenetic mechanisms that regulate the pseudomalignant and invasive behavior of the placenta in physiological and pathological pregnancies. This study was carried out in pregnant women with physiological pregnancy in three different trimesters: first, second, and third trimester and pregnant women with preeclampsia. Based on the lower levels of folic acid observed in preeclampsia (22), we studied the effect of these folic acid levels on various epigenetic mechanisms including global DNA methylation, Long INterspersed Element-1 (*LINE1)* promoter region methylation, and expression of DNMTs in different groups and preeclampsia. Additionally, the effect of folic acid supplementation on the invasion character, mRNA expression, and methylation of various genes were studied in first-trimester extravillous trophoblast transformed cell line (HTR-8/SVneo) and choriocarcinoma cell line JEG-3.

### Increased Global DNA Methylation Is Associated With Advancing Gestation and Preeclampsia Development

**Figure 2:**
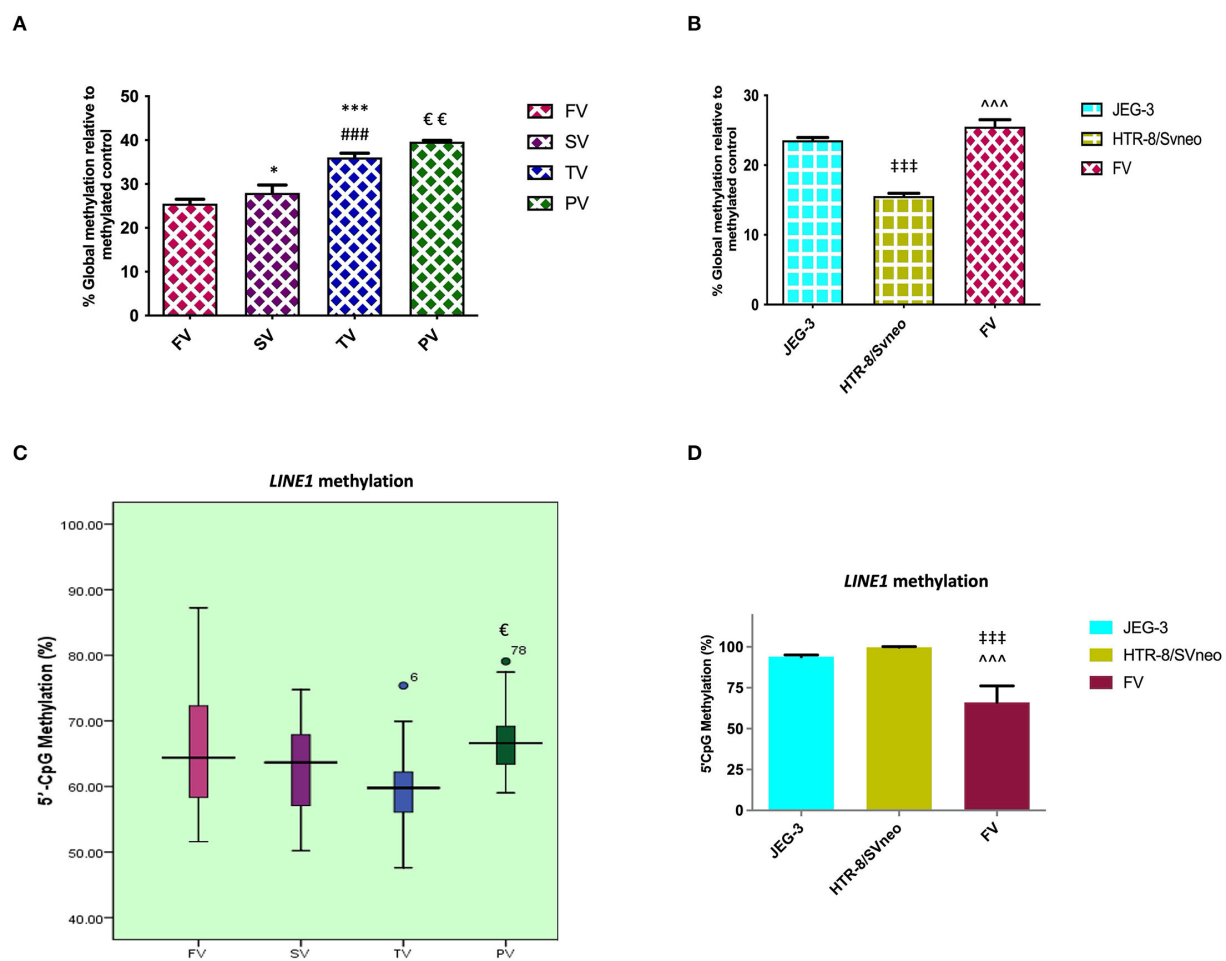
Percentage of relative global methylation and promoter region methylation for *LINE1*, **(A, B)** relative percentage global methylation **(A)** among different villi samples **(B)** among different placental cells viz JEG-3, HTR-8/Svneo, and first trimester villi. **(C, D)** Graphical representation of 5'CpG methylation (%), **(C)** among different villi samples **(D)** among different placental cell lines viz JEG-3, HTR-8/Svneo, and first trimester villi. The data are expressed as mean ± SEM. FV, SV, TV, and PV -first trimester, second trimester, third trimester, and preeclampsia placental villi samples respectively. [*p < 0.05, ***p < 0.001 with respect to FV, ^*###*^*p* < 0.001 with respect to SV and ^€^*p* < 0.05, ^€€^*p* < 0.01 with respect to TV] [^‡‡‡^*p* < 0.001 with respect to JEG-3, ^∧∧∧^*p* < 0.001 with respect to HTR-8/SVneo].

At the genomic level, global transcription is regulated by the overall degree of methylation of a genome, which includes CpG methylation both at intergenic levels as well as gene-specific promoter region methylation. Comparing the relative percentage of global DNA methylation in normal placentas of three different trimesters, we found global DNA methylation to increase with gestation, from 25.5% in the first-trimester to 27.99% (*p* < 0.05) in second- trimester and finally to 36.07% (*p* < 0.001) at full term. There was an increase in percentage global DNA methylation to 39.6%, *p* < 0.01, in preeclamptic placenta compared to their gestation age-matched control placentas as shown in Figure 2A.

Global methylation was also found to be significantly different in different placental cells, with the least global methylation in HTR-8/SVneo (15.57%, *p* < 0.001) compared to JEG-3 cells (Figure 2B).

### *LINE1* Promoter Region Shows Hypermethylation in Preeclampsia

*LINE1* constitutes almost 17–25% of the human genome with up to 600,000 copies per genome. CpG sites in LINEs comprise a big portion of the human genome, these are largely methylated in normal somatic tissue. Since these repeats make up a considerable portion of the human genome, the degree of methylation in these repeats comprises a significant portion of global methylation (28). However, other repeat elements and gene-specific promoter methylation are also important in determining total genomic methylation. In our study, MS-HRM analysis for the *LINE1* promoter region in the preeclamptic placenta showed a higher methylation by 7% (*p* < 0.05) in the *LINE1* promoter as compared to the normal third trimester. However, MS-HRM detected a nonsignificant change in methylation in normal gestational placental groups (Figure 2C).

*LINE1* promoter was detected to be highly methylated in HTR-8/SVneo and JEG-3cells as compared to normal first-trimester placental villi (Figure 2D), the average methylation percentage being 34 and 28 % higher, respectively (*p* < 0.001).

### mRNA Expression of *DNMT*s Is Deregulated in Preeclampsia

**Figure 3:**
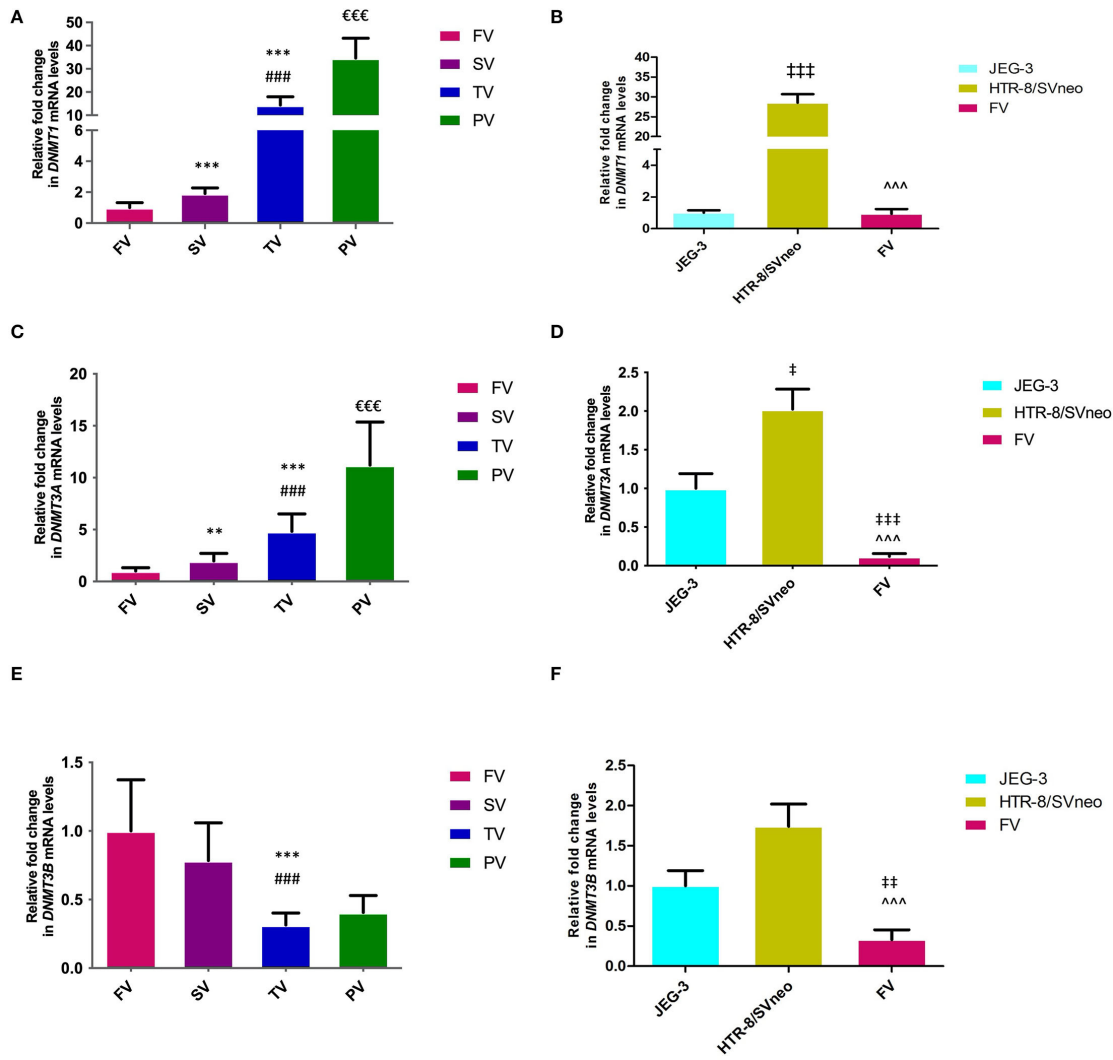
qRT-PCR analysis of DNMTs normalized with GAPDH. **(A, B)** Relative fold change in *DNMT1* mRNA levels, **(C, D)** relative fold change in *DNMT3A* mRNA levels **(E, F)** relative fold change in *DNMT3B* mRNA levels **(A, C, E)** among different villi samples **(B, D, F)** among different placental cells viz JEG-3, HTR-8/Svneo and first trimester villi. The data are expressed as mean fold change ± SEM. FV, SV, TV, and PV -first trimester, second trimester, third trimester, and preeclampsia placental villi samples respectively. [**p < 0.01, ***p < 0.001 with respect to FV, ^*###*^*p* < 0.001 with respect to SV, ^€€€^*p* < 0.001 with respect to TV], [^‡^*p* < 0.05, ^‡‡^*p* < 0.01, ^‡‡‡^*p* < 0.001 with respect to JEG-3 and ^∧∧∧^*p* < 0.001 with respect to HTR-8/SVneo].

We estimated the levels of expression for DNMT1, DNMT3a, and DNMT3B in different pregnancy groups. The mRNA expression of *DNMT1* was found to vary significantly within the different placental groups. In normal placental groups, there was a 2-fold increase in the second trimester relative to the first trimester, followed by a further 7.5-fold increased expression in the third- trimester relative to the second trimester (*p* < 0.001). Preeclamptic placenta revealed a significant increase of 2.4-fold (*p* < 0.001) expression of *DNMT1* relative to the third-trimester placenta (Figure 3A). The expression of *DNMT1* was found to be 28- to 30-fold higher in HTR-8/SVneo cells (*p* < 0.001) in comparison to JEG-3 cells and normal first-trimester villi, as shown in Figure 3B.

Normal gestational pregnancy was found to be accompanied by a consistent increase in the *DNMT3A* level in placental villi groups (Figure 3C). As observed by a 2-fold (*p* < 0.01) increase in the second-trimester and 4.8-fold (*p* < 0.001) increase in the third-trimester, relative to first-trimester group. The preeclampsia group showed a further increase of 2.3-fold (*p* < 0.001) relative to the normal third-trimester group. A comparison of the *DNMT3A* expression in placental derived cell lines and first trimester villi (Figure 3D) showed a 2-fold and 16.8-fold lower expression in JEG-3 and normal first trimester villi, relative to HTR-8/SVneo cells (*p* < 0.05 and *p* < 0.001, respectively).

On accessing the mRNA expression of *DNMT3B* in different placental groups, a decreased expression of 3.2 and 2.4-fold was observed in term placenta (*p* < 0.001), relative to first- and second-trimester placental villi groups respectively (Figure 3E). The relative *DNMT3B* expression was found to vary non-significantly between the two placental cell lines, however, both the cell lines showed higher *DNMT3B* mRNA levels in comparison to normal first trimester villi, with 3.03-fold (*p* < 0.01) and 5.3-fold (*p* < 0.001) increase in JEG-3 and HTR-8/SVneo cells, respectively (Figure 3F).

### Correlation Analysis of Folate Levels, Global DNA Methylation, *LINE1* Promoter Methylation, and mRNA Expression of Various DNMTs, Among Different Categories

To find the relation between various molecular aspects of our study, we have used Pearson's correlation analysis. Pearson's correlation analysis is a statistical relationship, or association, between two continuous variables. It was used to compare the mean data between two particular sets of experiments among groups. In our analysis, we have used Pearson correlation coefficient “r” value to define the relation as, if r <0.49: low relation, r = 0.5 to 0.7: moderate relation and r = 0.7 to 1: high relation.

In this study, on Pearson correlation analysis, among placental villi groups (normal first-, second- and third- trimester placenta and preeclampsia), revealed a significant positive and negative correlation between mRNA expression of *DNMT1* vs. *DNMT3A* (r = 0.99; *p* < 0.001) and vs *DNMT3B* (r = −0.77), respectively. Global methylation also showed a significantly higher positive correlation with mRNA expression level of *DNMT1* (r = 0.9; *p* < 0.05) and *DNMT3A* (r = 0.87; *p* < 0.05), while it was negatively correlated to mRNA expression of *DNMT3B* (r = 0.83, *p* < 0.05). Pearson correlation analysis showed a moderate correlation between *LINE1* promoter methylation and *DNMT3B* expression (r = 0.5). Within placental villi groups, almost no correlation was observed between *LINE1* promoter methylation and global DNA methylation. The folate level with different parameters showed a moderate negative correlation with *DNMT1, DNMT3A*, global DNA methylation, and *LINE1* promoter methylation, as shown in Table 1.

**Table 1:** Correlation analysis of mRNA expression of various DNMTs, folate level, and global methylation level among placental groups (normal first, second and third trimester, and preeclampsia).

	**Folate level**	**Global methylation**	***LINE1* methylation**	***DNMT1* mRNA expression**	***DNMT3A* mRNA expression**	***DNMT3B* mRNA expression**
Folate level	1	−0.404	−0.56	−0.53	−0.53	−0.09
Global methylation	−0.404	1	−0.006	0.901^*^	0.88^*^	−0.83^*^
*LINE1* methylation	−0.568	−0.006	1	−0.189	−0.222	0.528
*DNMT1* mRNA expression	−0.538	0.901^*^	−0.189	1	0.99^***^	−0.771
*DNMT3A* mRNA expression	−0.537	0.878^*^	−0.222	0.99^***^	1	−0.765
*DNMT3B* mRNA expression	−0.093	−0.832^*^	0.528	−0.771	−0.765	1

* *p < 0.05*,

*** *p < 0.001*.

### Effect of Folic Acid Supplementation on the Invasive Potential of Various Trophoblasts

Folic acid is a key source of the one-carbon group required to methylate DNA. DNA methylation is an epigenetic modification critical to the normal development of the placenta and regulation of its pseuduomalignant/invasive nature. In this context, we intended to find the role of folic acid in regulating the pseudomalignant/invasive nature of the placenta. Therefore, the effect of folic acid on the invasive potential of various trophoblasts was studied by supplementing folic acid to HTR-8/SVneo and JEG-3 trophoblast cell lines at two different concentrations i.e., 10^−7^M and 10^−4^M using matrigel based invasion assay. Among these two concentrations, 10^−7^M represents the concentrations in the physiological range (400–600 μg/day) and 10^−4^M represents a much higher concentration than the physiological range. The cells were treated for 48 h and allowed to invade across the Matrigel matrix coated PET membrane of cell culture inserts.

**Figure 4:**
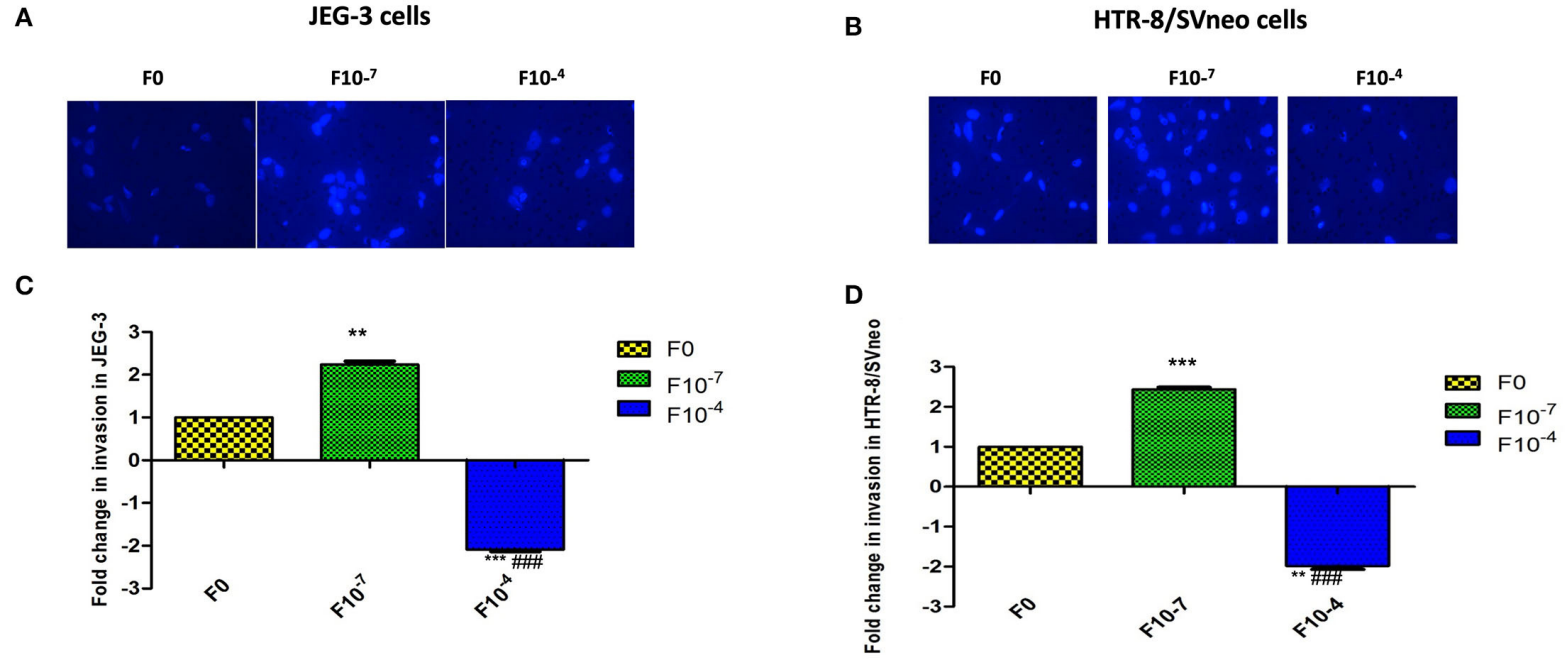
Invasion assay in trophoblast cell lines supplemented with folic acid. **(A, B)** JEG-3 cells **(A, C)** Hoechest 33342 staining of invaded cells. **(B, D)** Fold change in invasion in JEG-3 and HTR-8/SVneo cells after 48 h of treatment. F0-without folic acid supplementation, F10-^7^ and F10-^4^ folic acid supplementation at the concentration of 10-^7^ and 10-^4^ M, respectively. The data are expressed as mean ±SEM of three experiments. **p < 0.01 and ***p < 0.001 with respect to the F0 control and ^*###*^*p* < 0.01 with respect to F10-^7^ treated cells.

*In vitro* invasion assay showed a dose-dependent effect of folic acid supplementation on the invasiveness of both cell lines. In the JEG-3 cell line, there was a significant increase (*p* < 0.01) in invasion with 2.24-fold higher invasive potential at 10^−7^ M folic acid treatment with respect to untreated cells while treatment of folic acid at a higher concentration of 10^−4^M resulted in a significant (*p* < 0.01) decrease in invasion by 2.08-fold. Thus, showing a more significant difference in invasiveness at two different concentrations of folic acids (*p* < 0.001) (Figures 4A,B).

Studying the effect of folic acid treatment in the HTR-8/SVneo cell line revealed that the intrinsic invasive potential of HTR-8/SVneo was much higher (6.4-fold, *p* < 0.001) than the JEG-3 cell line. At 10^−7^M folic acid supplementation, there was a 2.4-fold (*p* < 0.001) increase in the invasive potential of the HTR-8/SVneo cell line with respect to control cells. There was a decrease in the invasion at 10^−4^M folic acid treatment by around 2-fold (*p* < 0.01) as compared to control (Figures 4C,D).

### Estimation of Folic Acid Levels in Trophoblast Cell Lines

Folic acid levels were estimated in JEG-3 and HTR-8/SVneo cell lines and cytotrophoblasts with and without folic acid supplementation. It was estimated by a microbiological assay using *Lactobacillus casei*. Folic acid levels are expressed as mean ±SD in each group.

**Figure 5:**
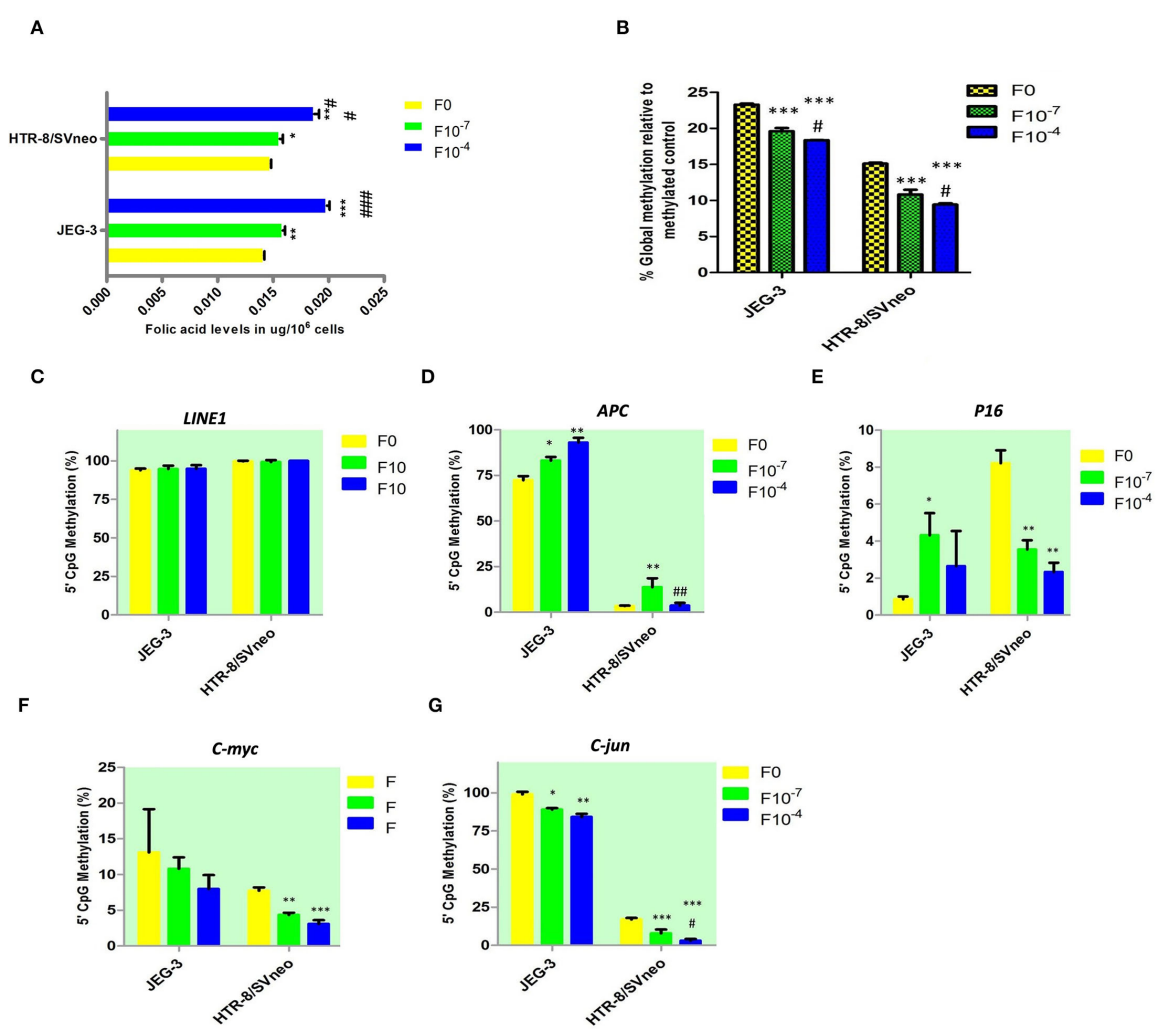
Effect of folic acid supplementation on DNA methylation in JEG-3 and HTR-8/Svneo cell lines. **(A)** Relative folic acid levels after folic acid supplementation **(B)** Percentage global methylation upon folic acid supplementation **(C–G)** Effect of folic acid supplementation on 5'CpG Methylation (%) in **(C)** *LINE1* promoter region, **(D)** *APC* promoter region, **(E)** *P16* promoter region, **(F)** *c-myc* promoter region, and **(G)** *c-jun* promoter region. F0- without folic acid supplementation, F10^−7^and F10^−4^ - folic acid supplementation at the concentration of F10^−7^ and F10^−4^ M respectively. The data are expressed as mean ±SEM of three experiments. *p < 0.05, **p < 0.01, ***p < 0.001 with respect to the F0 control and ^#^*p* < 0.05, ^*##*^*p* < 0.01, ^*###*^*p* < 0.001 with respect to the F10^−7^ treated cells.

Folic acid levels were found to be equal in JEG-3 and HTR-8/SVneo cell lines before the supplementation of folic acid (Figure 5A). The folic acid supplementation resulted in a dose-dependent increase in folic acid levels in both JEG-3 and HTR-8/SVneo cells. At 10^−4^M concentration, the levels of folic acid in JEG-3 cells were 0.02 μg/10^6^ cells (*p* < 0.001), while that in HTR-8/SVneo cells, they were 0.019 μg/10^6^ (*p* < 0.001), as compared to control cells (0.014 μg/10^6^ cells).

### Folic Acid Supplementation Alters DNA Methylation in Trophoblast Cells

Folic acid is a known mediator in DNA methylation. To elucidate the molecular mechanism of the changes induced in invasive potential of trophoblasts and regulation of pseudo-malignant nature of placenta, by folate supplementation, we analyzed the effect of folic acid supplementation on DNA methylation at the gene promoter level, global methylation, and LINE1 methylation levels.

#### Effect of Folic Acid Supplementation on Global Methylation

Global DNA methylation was measured at all CpGs irrespective of their position in the genome. Elucidating the role of folic acid in regulating placental global DNA methylation showed that folic acid treatment induces a dose-dependent decrease in global methylation in both cell lines. The percentage of global methylation relative to methylated control was 23.3 ± 0.15% and 15.1 ± 0.15% in JEG-3, HTR-8/SVneo cell lines, without folic acid supplementation. It decreased by 3.7% and 4.9% (*p* < 0.001) with 10^−7^M and 10^−4^M folic acid treatment, respectively, when compared to the untreated JEG-3 cells. The treated HTR-8/SVneo cells depicted a slightly higher decrease in % methylation, than treated JEG-3 cells. It decreased by 4.3% and 5.7% (*p* < 0.001) at 10^−7^M and 10^−4^M folic acid treatment with respect to the control HTR-8/SVneo cells (Figure 5B).

#### Effect of Folic Acid Supplementation on *LINE1* Promoter Methylation

MS-HRM detected hypermethylation in the *LINE1* promoter region in JEG-3 and HTR-8/SVneo cells, with no significant change induced in methylation level upon folic acid treatment in any of these cells types (Figure 5C).

#### Effect of Folic Acid Supplementation on Gene-Specific Promoter Methylation

We studied the effect of folic acid supplementation on the gene promoter region methylation in placental cell lines, viz JEG-3 and HTR-8/SVneo cells. For this purpose, we selected various categories of genes that are known to play role in regulating pseudomalignant/invasive nature of the placenta, based on the available literature and our previous studies. The various types of the genes that we studied were tumor suppressor genes (*RASSF1A, P16, RB1, PRKCDBP, APC*), oncogenes (*c-myc, c-jun, VEGF, EGFR*), telomerase (*hTERT*), and matrix metalloproteinases and their inhibitors (*MMP2, MMP9, TIMP1, TIMP2*).

Among the tumor suppressor genes, folic acid supplementation induced a significant change in promoter methylation of *APC* and *P16*. Promoter methylation was found to be significantly increased at APC promoters with the folic acid treatment increasing in JEG-3 cells by 1.14 fold (*p* < 0.05) and 1.28 fold (*p* < 0.01) at 10^−7^M and 10^−4^M folic acid treatment, respectively, compared to untreated control cells, while in HTR-8/SVneo cell APC methylation increased significantly by 4.1-fold at 10^−7^M only (Figure 5D). Surprisingly, folic acid-modified the *P16* promoter methylation (Figure 5E) in a reverse manner between choriocarcinoma cell line, JEG-3, and normal first- trimester cell line HTR-8/SVneo. *P16* promoter methylation increased in JEG-3 cells by 5 fold (*p* < 0.05) at 10^−7^M folic acid treatment compared to untreated cells, while in HTR-8/SVneo cells folic acid was found to decrease the *P16* methylation by 2.4 fold (*p* < 0.01) at 10^−7^M folic acid treatment and by 3.5 fold (*p* < 0.01) at 10^−4^M folic acid treatment.

Among the studied oncogenes, *c-myc* and *c-jun* showed a dose-dependent decrease in methylation in trophoblast cell lines. *C-myc* promoter methylation (Figure 5F) decreased in HTR-8/SVneo cells by 1.8 fold (*p* < 0.01) at 10^−7^M treatment and by 2.5 fold (*p* < 0.01) at 10^−4^M treatment relative to untreated cells. In JEG-3 cells, *c-jun* promoter methylation decreased by 1.1 fold (*p* < 0.05) and 1.2 fold (*p* < 0.15) at 10^−7^M and 10^−4^M folic acid treatment, respectively, relative to untreated cells (Figure 5G), while in HTR-8/SVneo cells *c-jun* promoter methylation decreased by 2.14 fold at 10^−7^M and 5.5 fold at 10^−4^M treatment of folic acid (*p* < 0.001), relative to untreated cells (Figure 5G).

MS-HRM detected a completely unmethylated promoter region in *hTERT* in JEG-3 and HTR-8/SVneo cells, with no change induced in methylation status in these cells by folic acid supplementation. The folic acid supplementation was not found to induce any significant change in promoter region methylation in *MMP2 and MMP9* and their inhibitors *TIMP1* and *TIMP2* promoters in any cell type.

### Folic Acid Supplementation Alters mRNA Expression in Trophoblast Cells

Further to study the transcriptional profile of various genes under study, upon folic acid supplementation, we quantified the mRNA levels of various studied genes in JEG-3 and HTR-8/SVneo cell lines after giving folic acid treatment at 10^−7^ and 10^−4^ M.

**Figure 6:**
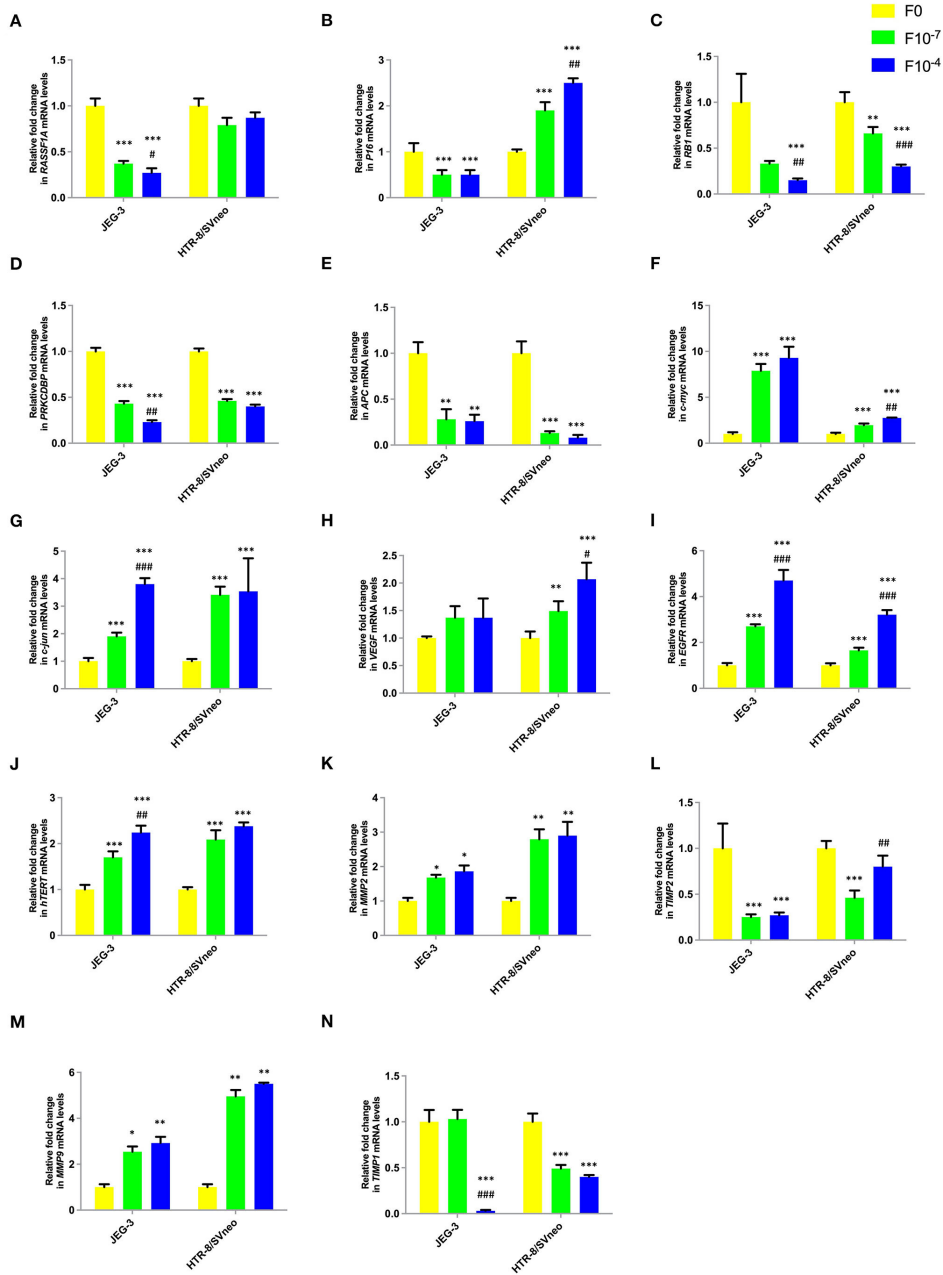
Effect of folic acid supplementation on mRNA levels of specific genes in JEG-3 and HTR-8/Svneo cell lines. Relative fold in mRNA levels of levels of **(A)** *RASSF1A* **(B)** *P16* **(C)** *RB1* **(D)** *PRKCDBP* **(E)** *APC* **(F)** *c-myc* **(G)** *c-jun* **(H)** *VEGF* **(I)** *EGFR* **(J)** *hTERT* **(K)** *MMP2* **(L)** *TIMP2* **(M)** *MMP9* **(N)** *TIMP1* F0- without folic acid supplementation, F10^−7^and F10^−4^ - folic acid supplementation at the concentration of F10^−7^ and F10^−4^ M, respectively. The data are expressed as mean ±SEM of three experiments. *p < 0.05, **p < 0.01, ***p < 0.001 with respect to the F0 control and ^#^*p* < 0.05, ^*##*^*p* < 0.01, ^*###*^*p* < 0.001 with respect to the F10^−7^ treated cells.

Folic acid treatment was found to significantly decrease the mRNA levels of studied tumor suppressor genes, *RASSF1A, RB1, PRKCDBP*, and *APC* either in one or both cell lines in a dose-dependent manner. However, folic acid was found to induce a reverse trend in mRNA levels in JEG-3 and HTR-8/SVneo cells in the case of P16 as shown in Figures 6A–E.

Folate was found to act as an inducer of studied oncogenes and hTERT expression. The expression of *c-jun, c-myc, EGFR*, and *hTERT* increased in a dose-dependent manner in both cell lines, while only HTR-8/SVneo cells showed such dose-dependent increased expression for *VEGF* (Figures 6F–J).

Folic acid supplementation altered the expression levels of MMPs and their inhibitors in a reverse trend in both cell lines. The expression of MMP2 and MMP9 increased, while that of TIMP1 and TIMP2 decreased in a dose-dependent manner in JEG-3 and HTR-8/SVneo cells (Figures 6K–N).

### Folic Acid Supplementation Alters mRNA Expression of *DNMT*s

**Figure 7:**
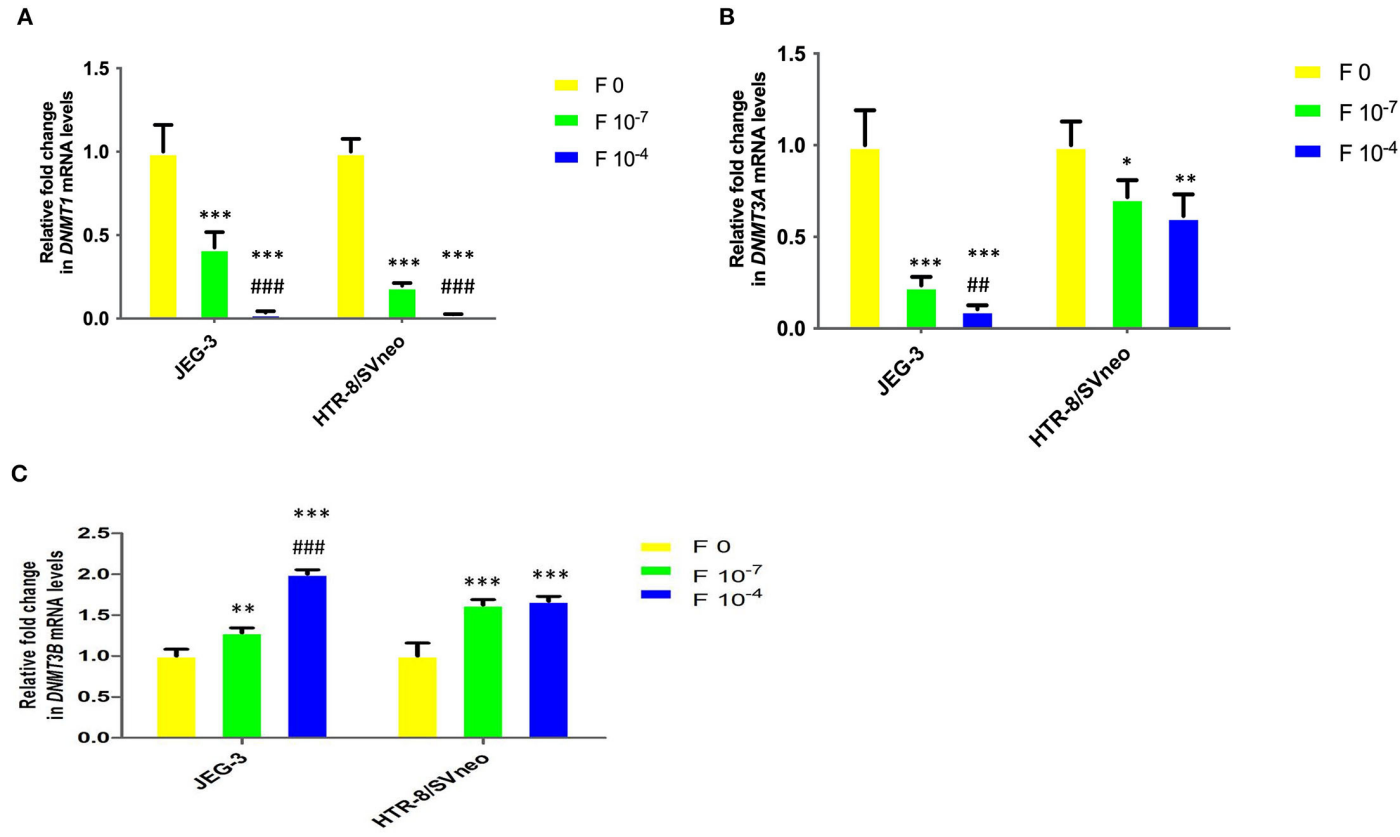
Relative fold change in mRNA levels of DNMTs upon folic acidsupplementation in JEG-3 and HTR-8/SVneo cells. **(A)** DNMT1, **(B)** DNMT3A, and **(C)** DNMT3B. F0- without folic acid supplementation, F10^−7^and F10^−4^ - folic acid supplementation at the concentration of F10^−7^ and F10^−4^ M, respectively. The data are expressed as mean fold change ±SEM of three experiments. *p < 0.05, **p < 0.01, ***p < 0.001 with respect to the respective F0 control and ^*##*^*p* < 0.01 and ^*###*^*p* < 0.001 with respect to respective F10-7 treated cells.

*DNMT1* mRNA level in placental cell lines showed a significant decrease upon the folic acid supplementation (Figure 7A). The decrease in *DNMT1* expression at 10^−7^M folic acid treatment was 2.3- and 5-fold in JEG-3 and HTR-8/SVneo cells, relative to their respective control cells. It was further reduced by 11.8-and 7.8-fold at 10^−4^M folic acid treatment, with respect to their respective 10^−7^M folic acid-treated cells.

Similar to that observed for *DNMT1*, folic acid inhibited the *DNMT3A* mRNA expression in cells by 4.2-and 9.4-fold (*p* < 0.001) in JEG-3 with respect to untreated control cells at 10^−7^M and 10^−4^ folic acid. In HTR-8/SVneo cells there was an almost equal decrease in *DNMT3A* relative expression (1.5-fold) at both the folic acid concentrations used (Figure 7B).

*DNMT3B* mRNA expression was found to be increased in both cell lines due to folic acid supplementation. In JEG-3 cells it increased by 1.3-fold (*p* < 0.01) and 2-fold (*p* < 0.001) at 10^−7^ M and 10^−4^ M folic acid concentration while in HTR-8/SVneo cells it increased by almost 1.6-fold (*p* < 0.001), with folic acid treatment, relative to their untreated control cells (Figure 7C).

### Correlation Analysis of Folate Levels, mRNA Expression of Various DNMTs, and Global Methylation Level in Trophoblast Cell Lines Upon Folic Acid Supplementation

On Pearson correlation analysis, between various DNMTs, folate level, and global methylation level and *LINE1* promoter methylation, in JEG-3 cells upon folic acid supplementation (Table 2), a strong negative correlation was observed between folate level vs *DNMT1*and *3A* (r > 0.78), while it was positive with *DNMT3B* mRNA expression (r = 0.99, *p* < 0.05). Folate vs *LINE1* promoter methylation was positively correlated (r = 0.86), while it was negatively correlated with global methylation (r = −0.87). A highly significant negative correlation was observed between global methylation and *LINE1* promoter methylation in JEG-3 cells upon folic acid supplementation (r = −1, *p* < 0.01), global methylation also showed a significant positive correlation with *DNMT3A* (r = 0.99, *p* < 0.05) (Table 2).

**Table 2:** Correlation analysis of mRNA expression of various DNMTs, folate level, and global methylation level in JEG-3 cells upon folic acid supplementation.

	**Folate level**	**Global methylation**	***LINE1* methylation**	***DNMT1* mRNA expression**	***DNMT3A* mRNA expression**	***DNMT3B* mRNA expression**
Folate level	1	−0.874	0.86	−0.94	−0.81	0.99^*^
Global methylation	−0.87	1	−1.00^**^	0.98	0.99^*^	−0.81
*LINE1* methylation	0.86	−1.00^**^	1	−0.98	−0.99^*^	0.79
*DNMT1* mRNA expression	−0.94	0.98	−0.98	1	0.96	−0.89
*DNMT3A* mRNA expression	−0.81	0.99^*^	−0.99^*^	0.96	1	−0.73
*DNMT3B* mRNA expression	0.99^*^	−0.81	0.79	−0.89	−0.73	1

* *p < 0.05*,

** *p < 0.01*.

In the case of HTR-8/SVneo, upon folic acid supplementation (Table 3), the Pearson correlation analysis between DNMTs mRNA expression, folate level, global methylation, and LINE1 promoter methylation showed a higher negative correlation in the case of folate level vs. expression of DNMT1 and DNMT3A (r = −0.77 and −0.83, respectively), while it was positive in case of DNMT3B (r = 0.98). However, global methylation was significantly correlated with mRNA expression of DNMT1 and DNMT3A (r = 0.99 and 1, respectively, *p* < 0.05). Furthermore, a significant positive correlation was observed between mRNA expression of DNMT1 vs DNMT3A (r = 0.99, *p* < 0.05) (Table 3).

**Table 3:** Correlation analysis of mRNA expression of various DNMTs, folate level, and global methylation level in HTR-8/SVneo cells upon folic acid supplementation.

	**Folate level**	**Global methylation**	***LINE1* methylation**	***DNMT1* mRNA expression**	***DNMT3A* mRNA expression**	***DNMT3B* mRNA expression**
Folate level	1	−0.82	0.63	−0.77	−0.83	0.98
Global methylation	−0.82	1	−0.075	0.99^*^	1.00^*^	−0.9
*LINE1* methylation	0.63	−0.07	1	−0.003	−0.09	0.49
*DNMT1* mRNA expression	−0.77	0.99^*^	−0.003	1	0.99^*^	−0.87
*DNMT3A* mRNA expression	−0.83	1.00^*^	−0.09	0.99^*^	1	−0.91
*DNMT3B* mRNA expression	0.98	−0.9	0.49	−0.87	−0.91	1

* *p < 0.05*.

The Pearson correlation analysis was also done between DNMTs mRNA expression, folate level, and global methylation level, separately in each group included in this study, which showed a significant correlation between *DNMT3A* vs. *DNMT3B* in first trimester placental villi (r = 0.811; *p* < 0.05), maternal blood leukocyte groups, *viz*, first trimester (r = 0.78; *p* < 0.05), second trimester (r = 0.8; *p* < 0.05), third trimester (r = 0.921, *p* < 0.01) and in JEG-3 cells (r = 1; *p* < 0.001). Furthermore, significant negative correlation was observed between folate level and global methylation in JEG-3 cells (r = −0.87; *p* < 0.05) and HTR-8/SVneo cells (r = −0.79; *p* < 0.05). Folate level vs. global methylation also showed higher Pearson coefficient value in preeclamptic placental group (r = 0.93) and in all maternal blood samples (r > 0.9), except that in first trimester.

## Discussion

Placental development involves complex molecular events like proliferation, migration, and invasion of placental trophoblasts, (2, 29, 30), therefore, the placentas act as a pseudomalignant tissue. Recent studies suggest the pivotal role of altered DNA methylation in regulating invasive behavior of trophoblast cells similar to many cancers (31). Studies also demonstrated the disrupted trophoblast invasive and migratory potential by blocking DNA methylation in trophoblasts (32). Folic acid is involved in DNA synthesis, repair, and methylation and acts as a cofactor in certain biological reactions. It is essential for cell multiplication and differentiation processes (33), however, in the case of pregnancy and infancy it is especially important in aiding rapid cell division and growth. In concern of pregnancy, the importance of folic acid in the prevention of neural tube defects has long been known (34). However, folic acid may also have important roles in other physiological pathways involved in pseudo-malignant nature of placentation, needed for a successful pregnancy, including rapid proliferation and invasiveness (35). Folic acid supplementation is recommended for women both in pre-conception and post-conception periods (36, 37). Therefore, we aimed at analyzing the epigenetic regulation of pseudo-malignant nature of placentation *via* folate levels both *in vivo* and *in vitro*. This was carried out by estimating the global DNA methylation, *LINE1* methylation, and expression of DNMTs and their correlation with folate levels in placenta from different gestational stages and preeclamptic women and by analyzing the effect of folic acid supplementation in trophoblastic and choriocarcinoma cell lines, on the pseudo-malignant phenotype induced by folic acid at the molecular and epigenetic levels.

Total global methylation is attributed to gene-specific promoter methylation, and the methylation of the various repeat elements in the genome, among those *LINE1* methylations, is considered a significant contributor. DNA methylation is coordinated by a family of DNA methyltransferases (DNMTs) comprising *DNMT1, DNMT*-*3A*, and *-3B*. *DNMT1* is considered as the maintenance DNA methyltransferase, while *DNMT3A* and *DNMT3B* are known as the *de novo* methyltransferases. These methyltransferases are important in regulating CpG methylation. are all essential for mammalian development. In addition to CpG methylation, *DNMT-3A* is also able to induce methylation at CpA and CpT (38). However, in our study, we have only focused on the CpG promoter region and *LINE1* methylation, which are known to regulate placental development and function. *DNMT3A* and *DNMT3B* are involved in establishing gene-specific and *LINE1* methylation (39). Furthermore, the involvement of *DNMT1* in global methylation in placental trophoblasts is well documented (40). Considering the current knowledge, we have thus analyzed global methylation in reference to *LINE1* methylation and gene-specific promoter methylation and predicted the possible involvement of specific DNMTs in global methylation in different categories.

We observed an increase in global methylation with advancing gestation accompanied by an increase in mRNA expression levels of *DNMT1* and *DNMT3A (*correlation value “r” = 0.9, 0.87, respectively, *p* < 0.05), which supports the earlier documented gestational stage-dependent increase of methylation levels (41). Based on our observations, we suggest that the increased expression of both *DNMT1* and *DNMT3A* is associated with increased placental global methylation with advancing gestation. This is also supported by the previously reported role of both *DNMT1* and *DNMT3A* in global methylation (39).

This increased global methylation was not accompanied by the increase in *LINE1* methylation in normal gestational placental groups, thus showing the possible involvement of other repeat elements and gene-specific methylation in increased global methylation. Relative global hypomethylation in early pregnancy may be a phenomenon regulating higher invasion in the first-trimester placenta. However, we observed higher global methylation and increased *LINE1* methylation in association with the higher expression of *DNMT1* and *DNMT3A* in the preeclamptic placenta. These results are consistent with a previous study showing the increased global and *LINE1* methylation in correlation with increased expression of *DNMT1* in early-onset preeclampsia (42). In our previous study, we reported lower folate levels in preeclamptic placental tissue relative to the normal third-trimester group. Therefore, we suggest the regulation of global methylation *via DNMT1* and *DNMT3A* and their dysregulation in preeclampsia under lower folate levels. Folate deficiency mediated decrease in methyl group might result in the hypomethylation of the regulatory CpGs within the *DNMT1*and *DNMT3A*, leading to their overexpression and subsequent increased global DNA methylation. This is supported with the study by Kim et al. who showed the upregulation of DNMT as well as genomic DNA hypermethylation in the liver of adult rat due to a short-term folate-deficient diet (43). In this context, a recent meta-analysis also emphasized the importance of the supplementation of multivitamins containing folic acid during pregnancy in reducing preeclampsia risk (23).

Hypomethylation at the global level as observed in various invasive tumors supports our observation of global methylation in JEG-3 and HTR-8/SVneo cells, which explains the highly invasive phenotype promoted by lower DNA methylation (44) in normal first trimester placental tissue. HTR-8/SVneo cells show much higher invasive potential relative to JEG-3 cells in culture, in matrigel assay (45), which might be due to the relatively lower global methylation level of HTR-8/SVneo cells with their higher invasive potential. Hence, the gestation associated increased global methylation might be due to increased expression of *DNMT1* and *DNMT3A*, and abnormally high DNMTs expression in association with folate deficiency results in the abnormal global methylation in preeclampsia.

Folic acid was found to induce a dose-dependent effect on the invasive potential of trophoblast cells. Folic acid supplementation at 10^−7^ M was found to increase the invasive potential of trophoblasts by more than 2-fold, showing invasion enhancing effect of folic acid at physiological concentration. A dose-dependent increase in invasive potential has earlier also been observed in extravillous trophoblasts from first-trimester placental explants at folic acid concentrations of 10^−6^ M, 10^−8^ M, and 10^−10^ M which represent physiological ranges (17). Furthermore, treatment with the anti-folate drug methotrexate in ectopic pregnancy has been shown to reduce placental growth and trophoblast invasion due to reduced trophoblast proliferation (46). Thus, we propose that at physiological concentrations, folic acid is able to increase trophoblast invasion, and therefore, play a key role in the regulation of trophoblast invasion, which is a crucial part of placental development, while at very high concentration, it reduces the invasion drastically. This biphasic nature of the response to folic acid supplementation, therefore, emphasizes the importance of careful evaluation of concentration before implementation of supplementation during pregnancy. This issue was also highlighted in a Cochrane review of micronutrient supplementation in pregnancy (47). Concentrations higher than the current daily recommendation of 400 μg may be harmful to placental development. Specific treatment with excess folic acid also results in reduced cellular viability in the villous trophoblast (18). Although too little folic acid results in nervous tissue damage, as is accepted by the scientific community in regard to neural tube defects, a massive dose of folic acid in *utero* has been correlated with the development of autism in children (48).

Investigating the molecular and epigenetic bases of the possible changes induced by folic acid supplementation on the invasive potential of trophoblasts showed a decrease in global methylation in JEG-3 and HTR-8/SVneo cells in a dose-dependent manner. Furthermore, folic acid treatment was observed to decrease the mRNA expression of *DNMT1*, which might be the possible reason for the reduced global methylation level. Since *DNMT1* expression is known to be regulated by promoter methylation in the placenta (40) and supplementation of folate may be inducing promoter methylation of *DNMT1*, resulting in its decreased mRNA expression. Recent data have implicated a role for the downregulation of *DNMT1* in the invasive and migratory potential of some cancer cells. Inhibition of the *DNMT1* activity in prostate cancer-derived PC3 cells enhanced invasiveness and migratory capacity (44). However, global hypomethylation in the absence of *DNMT1* down-regulation has been observed in human cytotrophoblast stem cells, suggesting that *DNMT1* down-regulation is not an absolute requirement for genomic hypomethylation in all instances (40), therefore highlighting the role of other DNMTs in global hypomethylation of the placenta. This is further supported by our observation that downregulation of *DNMT3A* upon folic acid treatment and a significantly strong correlation between global methylation vs *DNMT3A* mRNA expression (r = 0.99, *p* < 0.05) upon folic acid treatment. From these observations, we suggest that *DNMT1* and *DNMT3A* play a combined role in the down-regulation of the observed decrease in global methylation of placental trophoblasts resulting in their increased invasive potential.

Furthermore, folic acid supplementation induced increased invasion was mediated by the decreased expression of tumor suppressor genes and tissue inhibitors of matrix metalloproteinases, increased expression of oncogenes, telomerase gene, and matrix metalloproteinases, which were observed to be regulated by CpG methylation in the case of many genes. In the case of tumor suppressor genes, folic acid supplementation, in general, induces promoter methylation in unmethylated promoters like APC and *P16*. Folic acid supplementation decreased methylation in *c-myc* and *c-jun*, thereby promoting the malignant phenotype of placental trophoblasts. Folic acid was observed to induce gene-specific positive and negative changes in promoter methylation and mRNA expression in our study, this is consistent with the earlier studies showing the dual effect of folic acid supplementation on the global gene expression in certain cancers (11, 12, 14). Additionally, folic acid dysregulation may affect gene expression also by inducing base substitution, DNA breaks, gene deletions, and gene amplification (49).

## Conclusion

**Figure 8:**
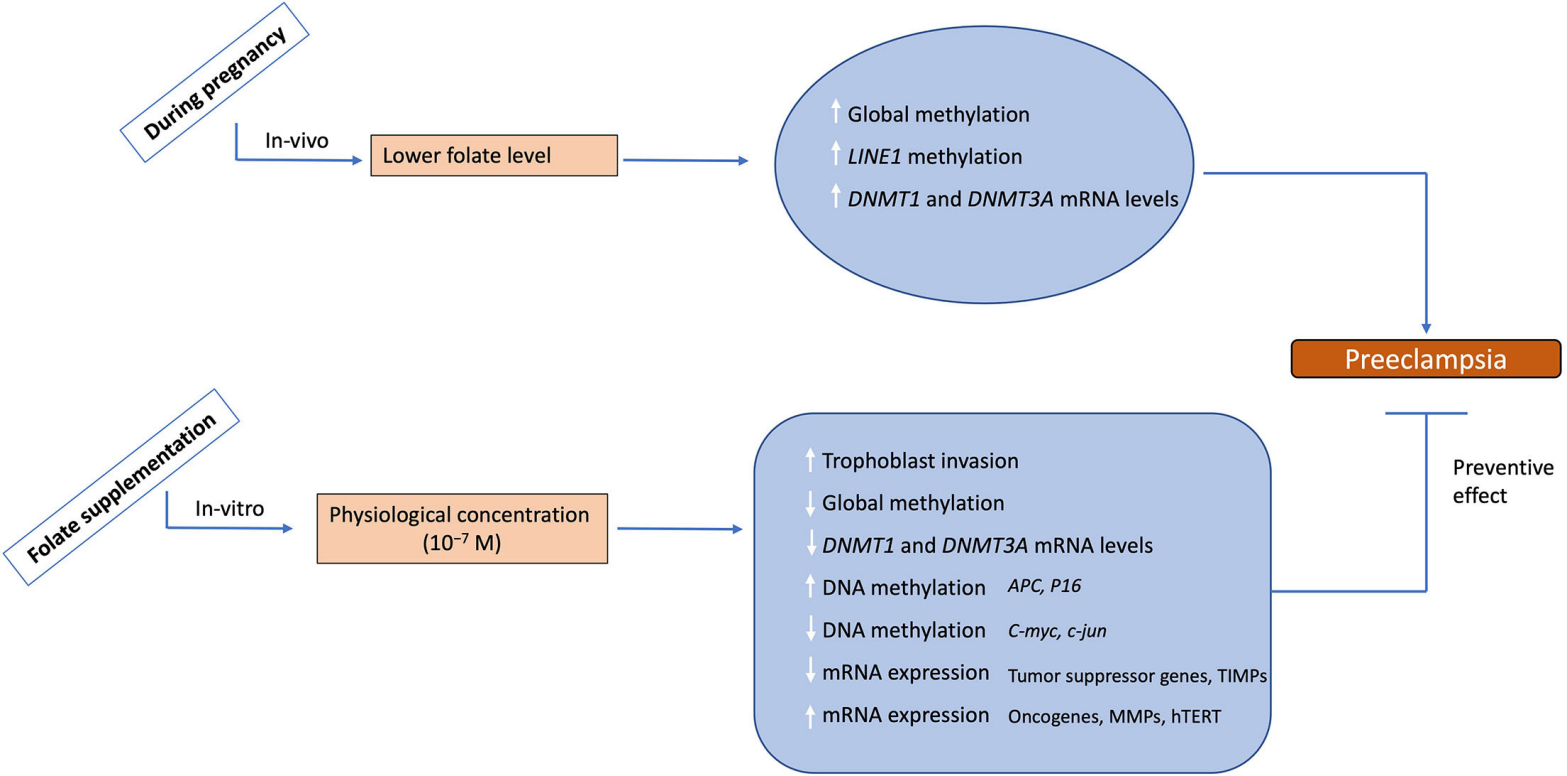
Summary of the study. During physiological pregnancy lower levels to folate are associated with increased global methylation, *LINE1* methylation and mRNA levels for *DNMT1* and *3A* leading to preeclampsia development. Folate supplementation at physiological levels leads to increased trophoblast invasion potential, decreased global methylation, decreased mRNA levels for DNMT1 and 3A, and alters DNA methylation/mRNA expression of various placental genes. This data demonestrates the possible role of folate supplementation in reversing the preeclampsia phenotype, hence suggesting a protective effect of folate supplementation. The upward arrows show an increase, while the downward arrows show the decrease in these parameters.

The major findings of this study are summarized and shown in Figure 8. Our results emphasize the role of epigenetic modifications, including DNA methylation in the regulation of differential gene expression involved in the invasive and malignant phenotype of placental development in normal pregnancy and dysregulation of these modifications in the development of preeclampsia. Furthermore, it highlights the impact of folic acid supplementation on trophoblast invasion, which is an important phenomenon regulating the outcome of pregnancy. The knowledge regarding the epigenetic regulatory mechanisms and their dysregulation in placental disorders, invasive changes mediated by folic acid supplementation can be used to develop therapeutic strategies for counteracting the clinical manifestations of pregnancy-related disorders like preeclampsia. The identification of these epigenetic mechanisms could provide novel targets for the diagnosis and treatment of both pathological pregnancies and fetal disorders

## Data Availability Statement

The original contributions presented in the study are included in the article/supplementary materials, further inquiries can be directed to the corresponding author.

## Ethics Statement

The studies involving human participants were reviewed and approved by Institute Ethics Committee (IEC), PGIMER. The patients/participants provided their written informed consent to participate in this study.

## Author Contributions

BR and JK designed the study and analyzed the data, and prepared the manuscript. BR performed the experiments. RB collected clinical data and helped in patient recruitment. AH helped in the analysis of methylation data. While all authors critically revised and approved the manuscript.

## Funding

This work was supported by the Indian Council of Medical Education and Research for funding this work (ICMR 5/10/FR/3/2010-RHN).

## Conflict of Interest

The authors declare that the research was conducted in the absence of any commercial or financial relationships that could be construed as a potential conflict of interest.

## Publisher's Note

All claims expressed in this article are solely those of the authors and do not necessarily represent those of their affiliated organizations, or those of the publisher, the editors and the reviewers. Any product that may be evaluated in this article, or claim that may be made by its manufacturer, is not guaranteed or endorsed by the publisher.
